# A Framework for Predicting X-Nuclei Transmitter Gain Using ^1^H Signal

**DOI:** 10.3390/tomography9050128

**Published:** 2023-08-24

**Authors:** Michael Vaeggemose, Rolf F. Schulte, Esben S. S. Hansen, Jack J. Miller, Camilla W. Rasmussen, Jemima H. Pilgrim-Morris, Neil J. Stewart, Guilhem J. Collier, Jim M. Wild, Christoffer Laustsen

**Affiliations:** 1 GE HealthCare, 2605 Brondby, Denmark; michael.vaeggemose@ge.com; 2 MR Research Centre, Aarhus University, 8200 Aarhus, Denmark; esben@clin.au.dk (E.S.S.H.); jack.miller@clin.au.dk (J.J.M.);; 3 GE HealthCare, 80807 Munich, Germany; schulte@ge.com; 4 POLARIS Group, University of Sheffield, Sheffield S10 2TN, UK; jhpilgrim-morris1@sheffield.ac.uk (J.H.P.-M.); neil.stewart@sheffield.ac.uk (N.J.S.); g.j.collier@sheffield.ac.uk (G.J.C.); j.m.wild@sheffield.ac.uk (J.M.W.)

**Keywords:** X-nuclei imaging, radio frequency setting, magnetic resonance imaging, sodium, carbon, xenon

## Abstract

Commercial human MR scanners are optimised for proton imaging, containing sophisticated prescan algorithms with setting parameters such as RF transmit gain and power. These are not optimal for X-nuclear application and are challenging to apply to hyperpolarised experiments, where the non-renewable magnetisation signal changes during the experiment. We hypothesised that, despite the complex and inherently nonlinear electrodynamic physics underlying coil loading and spatial variation, simple linear regression would be sufficient to accurately predict X-nuclear transmit gain based on concomitantly acquired data from the proton body coil. We collected data across 156 scan visits at two sites as part of ongoing studies investigating sodium, hyperpolarised carbon, and hyperpolarised xenon. We demonstrate that simple linear regression is able to accurately predict sodium, carbon, or xenon transmit gain as a function of position and proton gain, with variation that is less than the intrasubject variability. In conclusion, sites running multinuclear studies may be able to remove the time-consuming need to separately acquire X-nuclear reference power calibration, inferring it from the proton instead.

## 1. Introduction

In conventional proton MRI, essential acquisition parameters, including transmitter power, excitation frequency, and receiver frequency are typically automatically calibrated using vendor-supplied, automated prescan procedures [1]. Unfortunately, this process is not necessarily straightforward when scanning other nuclei, particularly for hyperpolarised (HP) experiments, where the signal changes during the experiment. The HP media signal is dramatically increased but is not in an equilibrium state and cannot recover polarisation after excitation. This permits fast acquisition schemes as signal relaxation does not occur [2]. The polarisation loss per excitation requires careful flip angle considerations either to preserve xenon gas polarisation (low flip angles) [3] or in the evaluation of pyruvate metabolism in hyperpolarised carbon [4].

Switching between proton and X-nuclei typically requires manually setting the transmit radiofrequency (RF) power and the X-nuclei centre frequency. While the chemical shift between the proton and X-nuclei is fixed for a given molecule of interest, it can be challenging to determine the multi-nuclear amplifier power levels required to achieve the prescribed flip angle. This is one factor among many required to perform hyperpolarised X-nuclei experiments reliably, including B_0_ shimming, the accurate estimation of the centre frequency, and setting analogue and digital receive gain. The ability to estimate the transmitted power levels conversion between the proton and X-nuclei is the focus of this study.

The required transmit RF power can be determined through various methods. Methods include calculating the flip angle from stimulated echoes from a series of RF pulses on a thermally polarised phantom [1,5]. This approach matches the maximum signal to a flip angle of 90° and sets 180° to be at twice the power. Other approaches include the use of a dual flip angle [6], multiple TRs [7], and the Bloch–Siegert phase shift [8,9] methods. In hyperpolarised gases, the inherent signal loss from the T_1_ relaxation and the RF excitation of magnetisation can be utilised for the estimation of the transmit gain (TG) [10,11,12].

RF coil loading is directly proportional to the frequency [13,14]. As such, ^129^Xe, ^13^C, and ^23^Na are ¼ as sensitive to coil loading and, therefore, less likely in need of considerable power adjustments as a similar coil design on ^1^H. This has led to the use of fixed transmitter power using phantoms or previously recorded in vivo values in hyperpolarised ^13^C experiments [15,16,17,18]. This practice indicates that TG is largely unaffected by patient loading and only in the most severe cases would require adjustments. Therefore, a simple relationship between the more sensitive ^1^H coils and the X-nuclei coils could be used for TG calibration. Implementations of nuclei and coil relationships may provide valuable information for automatic prescan methods and the clinical transition of X-nuclei examinations.

The aim of this study is to demonstrate and quantify the reproducibility of a simple method for the automatic setting of X-nuclei transmitter power levels based on prior proton settings.

## 2. Materials and Methods

### 2.1. Transmitter Gain

The RF excitation flip angle can be controlled by changing the transmitter gain (TG), here measured in dB, and the RF pulse duration [19]. The exact B1 delivered can be measured using the Bloch–Siegert phase shift approach. The approach measures a phase difference induced by an off-resonance pulse applied after excitation [8]. The measured phase difference is proportional to the square of the applied transmit B1 field [20], permitting the determination of the TG for an X-nuclei. Given the robustness of the Bloch–Siegert phase approach [20] in different anatomies, this approach was used throughout the paper for determining the correct RF power levels of the proton and three X-nuclei: sodium, xenon, and carbon.

### 2.2. Study Design

Sodium and carbon data were acquired at Aarhus University (Site A) and hyperpolarised xenon at the University of Sheffield (Site B). Sodium and xenon images were acquired in vivo and carbon images on phantoms.

Site A used a 3T MRI scanner (GE MR750, GE HealthCare, Waukesha, WI, USA) to acquire proton, carbon (^13^C), as well as sodium (^23^Na) data. A commercial Helmholtz coil pair (PulseTeq, Surrey, UK) was used for ^23^Na and a similar coil was used for ^13^C. The diameter of the coil loops was 20 cm. The system body coil was used for proton imaging.

In vivo proton TG was determined using the MR system auto-prescan [1]. X-nuclei TG and centre frequency calibration were performed using the Bloch–Siegert (BLOSI) method in a spoiled spectroscopy sequence [20] with a constant frequency shift of ±2000 Hz. Sequence parameters are presented as [Sodium, Proton, Carbon], respectively: TR = [250, 500, 1000] ms; TE = 4.75 ms; flip angle = 90°; FOV = [35, 48, 48] cm; [100, 10, 20] mm thickness; spectral sample points = [1024, 1024, 2048]; bandwidth = 5 kHz; BLOSI off-resonance pulses = [16, 16, 32]; RF-pulse type [slice-selective (soft), slice-selective (soft), frequency-selective (hard)]; and total acquisition time = [8, 16, 64] s. To avoid analogue-to-digital converter (ADC) signal overflow, proton analogue (R1) and digital receiver (R2) gains were manually set to 2 and 20, respectively. Sodium and carbon receive gains were set to R1 = 13 and R2 = 30, which is the maximum.

Sodium data were acquired in two steps. Five healthy subjects (mean age 34 (range: 26–42) years, weight 70 (45–94) kg, height 176 (163–190) cm; 60% male) were included to evaluate data acquired from the brain, heart, kidneys, and thigh muscles. Circadian (morning, noon, evening) reproducibility was evaluated in the kidneys of ten healthy subjects (aged 28.4 (21–36) years, weight 70.8 (56–94) kg, height 176 (163–190) cm, 50% male). All subjects gave written informed consent and local ethics committees approved the study (No. 1-10-72-210-21) and ClinicalTrials.gov (No. NCT05215938).

The subjects in this multi-organ study were scanned twice leading to a total of 10 scan sessions of proton and sodium images. Proton and sodium repeatability and variability were estimated on a circadian data initial scan (morning) and follow-up scans (noon and evening). An analysis of the flip angle variation was performed to evaluate potential confounders in proton and sodium imaging. The relationship between the TG and flip angle is given in Equation (1) [21], which can be used to determine the actual flip angle (Equation (2)) for a desired flip angle of 90°.

(1) $${TG}_{\theta }={TG}_{90{}^{\circ}}+20\cdot \log_{10}\left(\frac{90{}^{\circ}}{\theta }\right)\;$$

(2) $$\theta_{actual}=\frac{90{}^{\circ}}{10^{\left(\frac{{TG}_{\theta }-{TG}_{90{}^{\circ}}}{20}\right)}}$$

Assuming an average of the circadian TG is a good measure of the TG at the prescribed 90° flip angle, the equation can be rewritten to Equation (3):

(3) $$\theta_{actual}=\frac{90{}^{\circ}}{10^{\left(\frac{{TG}_{measured}-{TG}_{mean}}{20}\right)}}$$

**Figure A1:**
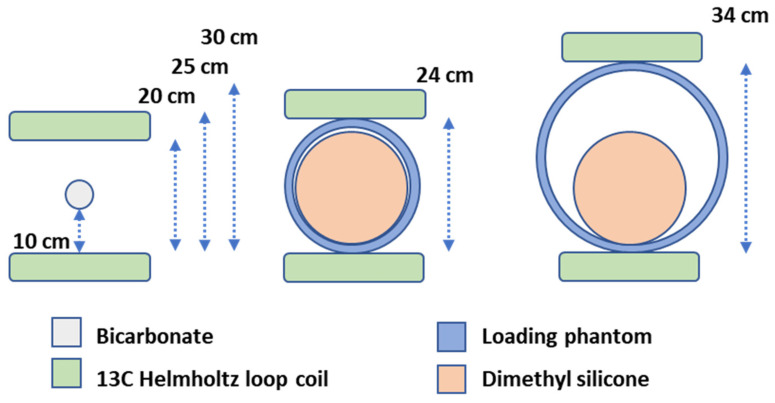
Phantom and coil placements in carbon transmit gain examinations. Bicarbonate point phantom with no proton loading placed in the centre between the two Helmholtz loop coils (**left**). Loading setup using head (**middle**) and body (**right**) load rings with the head dimethyl silicone ball inside. Distances between the Helmholtz loop coil elements are noted in cm. Three distances were evaluated in the bicarbonate setup (20, 25, and 30 cm).

The relationship between the proton and carbon TG was evaluated in phantoms. A dimethyl silicone phantom (DSP) containing natural abundance carbon [22] (GE HealthCare, Waukesha, WI, USA) was placed in a dielectric loading ring mimicking head and body loading (Figure A1). The coil distance dependency was evaluated using a 38 mm diameter spherical bicarbonate phantom containing a 1.0 M solution of ^13^C-bicarbonate sodium salt (85 mg/mL) in water. The phantom was placed 10 cm above the lower loop coil and the distance between the coil elements was increased using cushions from 20 cm to 30 cm in intervals of 5 cm. Scanning was repeated 5 times while resetting shimming of B0 between scans.

Site B used a 1.5T MRI scanner (HDx, GE HealthCare, Waukesha, WI, USA) to acquire proton MRI as well as hyperpolarised xenon (^129^Xe) data. A commercial quadrature vest coil (Clinical MR Solutions, Brookfield, WI, USA) was used for xenon and the system body coil for proton. As at site A, the proton TG was determined using the scanner auto-prescan procedure, and the BLOSI method was used for ^129^Xe with a frequency shift of ±2000 Hz. Sequence parameters for ^129^Xe were the following: TR = 75 ms; TE = 4.75 ms; flip angle = 10°; FOV = 40 cm; spectral sample points = 1024; bandwidth = 20 kHz; BLOSI off-resonance pulses = 2, averages = 1; pulse type frequency-selective (hard); and total acquisition time = 2 s.

Xenon data were retrospectively collected from 96 patients with a variety of lung disorders, including COPD, asthma, and lung disease post-COVID-19 infection, across 106 visits. These patients were recruited through studies approved by the local ethics committee that ran from 2020 to 2022. Patients were aged 61.6 (30.7–82.2) years, with weight 83.6 (41–130) kg, and height 168.6 (144.6–205.5) cm. The relationship between the proton and xenon TG values was tested using a simple linear regression model.

### 2.3. Statistics

The relationship between the RF transmit gain on the proton and the X-nuclei (sodium, xenon, carbon) was assessed by fitting a simple linear regression model to the transmit power gain in dB. Wilcoxon paired tests were performed for measured and predicted sodium TG values in each organ (kidney, heart, muscle, and brain). One-way ANOVA for multiple comparisons was performed on the repeated sodium measurements to establish circadian variance. F-tests compared the variation in the transmit gain and the actual flip angle of the proton, sodium, and xenon acquisitions. Statistical significance was defined as *p*-value below 0.05.

## 3. Results

### 3.1. Sodium

**Figure 1:**
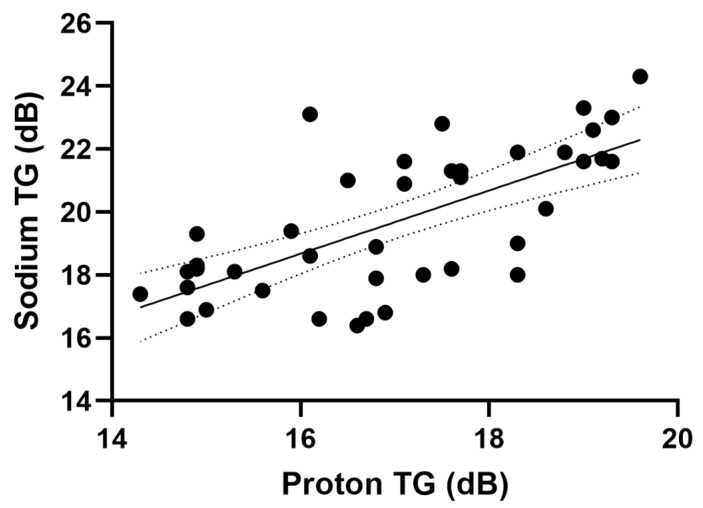
Proton and sodium transmit gain with a linear regression model fit.

Applying the linear regression model approximation to data pooled from the multi-organ data shows a relationship between the transmit gain of the sodium and proton (Figure 1).

From the results, we determined a relationship (R^2^ = 0.474, *p* < 0.001) between the proton and sodium TG as the following linear regression model.

(4) $$Sodium_{TG}=1.005\cdot Proton_{TG}+2.601$$

(5) $$Sodium_{TG}\approx Proton_{TG}+2.6$$

**Figure 2:**
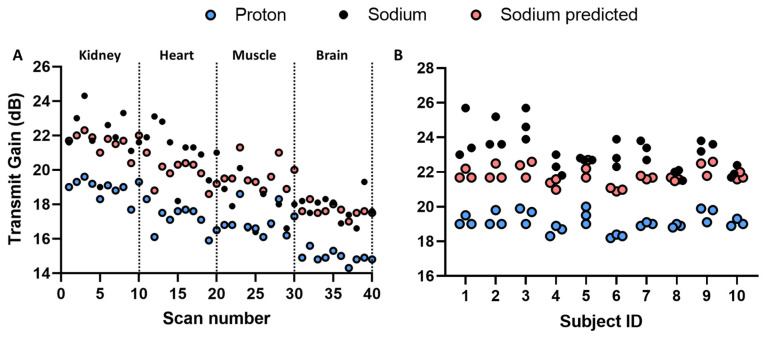
(**A**) Multi-organ measured and predicted transmit gain of proton and sodium with indication of anatomies of interest. (**B**) Circadian measured and predicted transmit gain of proton and sodium in kidney imaging.

All data points from the Site A sodium TGs comparison are shown in Figure 2. A decrease in the proton and sodium transmit gain in organs from the kidney to the brain can be seen, which corresponds to the decrease in loading. This is seen in both the acquired and predicted data. The predicted sodium transmit gain is significantly lower than the prescribed values in the heart (*p* = 0.04) and higher in the thigh muscle (*p* = 0.001). No significant difference was determined in the brain and kidney.

**Figure 3:**
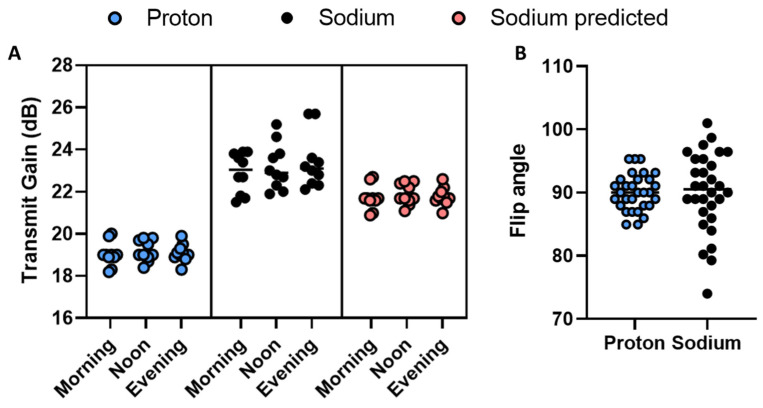
(**A**) Circadian measured and predicted proton and sodium transmit gain on a group level. (**B**) Actual flip angle spread of proton and sodium circadian measurements.

From the multi-organ study, we assumed that the sodium TG can be predicted from the proton scan. Evaluation of the model feasibility was performed by scanning the kidneys of 10 healthy subjects three times (morning, noon, and evening) within one day (Figure 2). Since each subject was scanned three times, a measure of variability can be determined (Figure 3).

There was no significant difference across time (morning, noon, and evening) with the measured proton or sodium TG, or the predicted sodium TG (via one-way ANOVA). A comparison of variation in the actual flip angle delivered shows a lower standard deviation in the proton transmit gain (Figure 3B). Defining the actual flip angle variation with 90° as a reference, calculated from the TG difference from the mean TG per subject (Equation (3)), we observed a larger variation in the sodium (90.33° ± 6.58°) compared to the proton (90.01° ± 2.90°). The difference in flip angle variance is significant (*p*-value < 0.001), indicating the proton TG to be a more robust measure than the sodium TG.

### 3.2. Xenon

**Figure 4:**
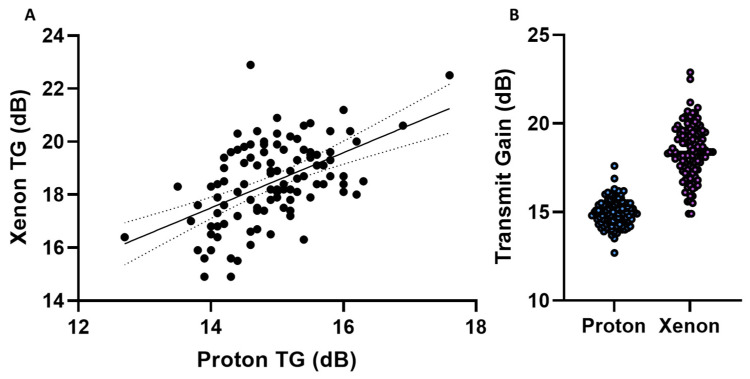
(**A**) Proton and xenon transmit gain with a linear regression model fit. (**B**) Proton and xenon transmit gain variation.

The proton and xenon transmit gain relationship was found via linear regression (Figure 4A) and approximated as follows:

(6) $${Xenon}_{TG}=1.041\cdot Proton_{TG}+2.926$$

(7) $${Xenon}_{TG}\approx Proton_{TG}+2.9$$

The R-squared value was 0.27, and the *p*-value was below 0.001. As with sodium, we evaluated the transmit gain variation of the proton and xenon (Figure 4B). The results show a significantly (*p* < 0.001) smaller variation in the proton TG (14.9 ± 0.8 dB) compared to the xenon TG (18.4 ± 1.5 dB), indicating the TG measurement for the proton to be a more robust measure than for xenon. By using the proton TG to determine the xenon TG, we can calculate the mean percentage relative difference in TG (for measurement vs. prediction) as follows:

(8) $${ratio}_{mean\;in\;\%}=\frac{1}{N}\sum \frac{{{Xenon}_{TG,predicted}-Xenon}_{TG,measured}}{{Xenon}_{TG,measured}}\cdot 100\%$$

This indicates an improvement in the xenon TG precision by a mean bias of 2.98 ± 6.87% and a reduction in variance by 1.74 dB.

### 3.3. Carbon

**Figure 5:**
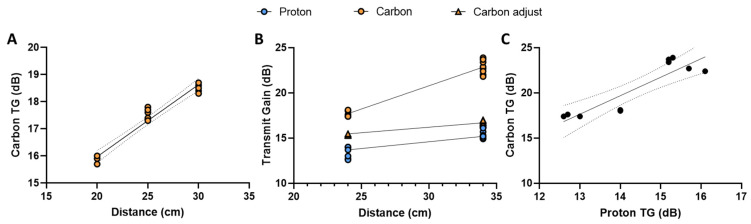
Proton and carbon transmit gain changes according to loading and distance. (**A**) Transmit gain changes according to changes in distance between the Helmholtz loop coil elements with a bicarbonate point phantom. (**B**) The transmit gain of proton and carbon in the head and body load with dimethyl silicone phantom. Carbon is shown with and without bicarbonate point phantom adjustment for distance between the Helmholtz loop coil elements in (**B**). (**C**) Proton and carbon transmit gain with a linear regression model fit.

Results from the Carbon-Proton phantom study are shown in Figure 5. The results display a mean value increase in the proton and carbon TG according to loading from 13.3 to 15.3 dB and 17.7 to 20.9 dB, respectively (Figure 5B). The transmit field varies across the distance between the Helmholtz loop coil elements in a manner that can be analytically known in detail [23]. Near the centre of the coil, the transmit field changes slowly as the first, second, and third derivatives of the spatial field are zero. Beyond this point, homogeneity is analytically approximated well by a first-order Taylor series [23]. This was estimated by determining the TG for a carbon point phantom at distances from 20 cm to 30 cm in increments of 5 cm (Figure 5A). Secondly, the TG changes were measured for the proton (head and body loading phantom) and the carbon (DSP) at 24 and 34 cm coil element distances (see Figure A1). The unadjusted linear relationship between the proton and carbon transmit gain, based on loading, was found via linear regression (Carbon_TG_ = 2.045 ∙ Proton_TG_ − 8.949, R^2^ = 0.81, *p*-value = 0.0004) (Figure 5C). Changing the loading to distance provides a linear relationship between the transmit gain and distance as shown in Figure 5A,B.

The relationship between the unadjusted carbon and distance can be linearly approximated as follows:

(9) $${Carbon}_{TG}=0.266\cdot distance+10.65$$

From the results, we achieve an R squared value of 0.95 with a *p*-value below 0.0001, with 95% confidence intervals of the slope and intercept being 0.231 to 0.301 dB∙cm^−1^ and 9.76 to 11.54 dB∙cm^−1^, respectively.

Distance correction is performed by combining carbon TG functions (loaded and unloaded) into a composite function (see Appendix A.2, Carbon composite functions). The composite transmit gain equation provides a linear relationship between the proton, carbon, and carbon-adjusted TG and can be determined as a function of distance as opposed to loading (Figure 5B).

(10) $${Proton}_{TG}=0.199\cdot distance+8.55$$

(11) $${Carbon}_{TG}=0.515\cdot distance+5.34$$

(12) $${Carbon}_{TG\_adjust}=0.137\cdot distance+12.07$$

The proton and carbon linear regressions had R-squared values of 0.81 and 0.95 with a *p*-value below 0.0001, respectively. The 95% confidence intervals of the proton and the adjusted carbon slopes are 0.1389 to 0.2594 dB∙cm^−1^ and 0.1154 to 0.1586 dB∙cm^−1^. The proton and adjusted carbon intercepts confidence intervals are 6.74 to 10.37 dB and 11.41 to 12.73 dB, respectively.

The proton and carbon (adjusted) TG slopes cannot be statistically rejected as equal given the overlapping confidence intervals. Therefore, we assume the slopes to be comparable and the change in the TG dependent on loading. The relationship between the carbon and proton TG can then be written as the difference in their intercepts:

(13) $${Carbon}_{TG}\approx Proton_{TG}+3.52$$

This provides a linear relationship between the proton and carbon equal to the proton TG with an offset.

## 4. Discussion

The study has investigated the validity of using a linear relationship between the transmitted power gain (TG) of proton and sodium, xenon, and carbon in order to predict the X-nuclear TG from proton automatic power calibration. This offers the potential of applying an automatically setting X-nuclei TG based on the previously acquired proton images alone. Three X-nuclei were evaluated; however, the approach can be applied to any nuclei.

The TG increased linearly with loading and indicates using the proton TG; as a predictive model, is reasonably accurately indicated by a low difference between the predicted and measured X-nuclei TG. Only minor variation in the model was seen when evaluating repeatability. Repeatability was not evaluated in hyperpolarised xenon examinations. Nevertheless, the mean relative difference between the measured and predicted xenon TG was 2.98 ± 6.87% over 106 examinations. Resulting in a flip angle difference of 0.15° ± 1.78° and 0.04° ± 0.86° for measured and predicted xenon, respectively. This difference is considered small and advocates for the application of an automated X-nuclei prescan option in hyperpolarised xenon gas imaging. This could improve workflow and, more importantly, reduce the dose of administered hyperpolarised gas. This is the first study presenting a framework for multiple X-nuclei with a special interest in hyperpolarised xenon-129 and carbon-13. In vivo results showed a small difference between the predictions and advocates for the application of an automated X-nuclei prescan option without the use of phantoms, calibration samples, or default values. To provide an overview, a tabular representation of the pros and cons compared to the literature is listed in Table 1.

The RF transmit (B_1_^+^) is determined by the delivered power of the RF amplifier, duration, and characteristics of the RF coil to produce the desired flip angles when exciting the nuclei spins. Furthermore, the transmitted power is proportional to the transmit frequency, coil loading (dimensions and dielectric properties), and distance to the imaging location. Higher frequencies require more RF power to achieve the same B_1_^+^ strength. The effect is caused by the shorter RF wavelengths, which produce higher power absorption. In the proton RF transmit, frequencies change from 64 MHz (1.5 T) to 128 MHz (3 T), whereas X-nuclei resonance frequencies are lower, prescribed by their gyro magnetic ratio. The difference in transmit power requirements is seen in the RF amplifiers, which are two separate modules depending on the target nuclei being a proton or X-nuclei. The maximum RF power for a proton on the system at site A is 30 kW on the proton and 8 kW for X-nuclei, which explains the lower transmit power gain in dB for the proton compared to the X-nuclei. Systems settings might vary slightly as acceptance levels, e.g., the X-nuclei on this system range from 7.2 kW to 8.2 kW. It is important to note that regular service could adjust these settings and thereby change the coil-specific TG settings. Furthermore, the reader should be aware of system conventions in the transmit gain settings. The system in this study measures the TG in centibel (cB), whereas RF measurements in the paper are reported in decibels (dB). Differences in vendor approaches to X-nuclei calibration should also be considered as some focus on voltage [24] and others on power. Even though the proportionality between the proton and X-nuclei may be different, a linear relationship should hold. The linear relationship should be determined as if a new coil were to be evaluated.

The efficiency of coils is often determined by the quality factor (Q-factor). The Q-factor is the ratio of stored and dissipated energy and changes according to loading [30]. The efficiency of the coil is determined as the ratio of the unloaded (no sample) and the loaded (sample) Q-factor. Hence, the sample size is an important factor in the determination of the optimal transmitted RF power. Larger samples will induce a need for a higher transmitted power as the distance between the coil elements increases. Surface and single loop coils are often used in X-nuclei data acquisition, as the design of these coils is relatively simple in construction, comparatively inexpensive, and can be used for a large variety of applications. A disadvantage is the radiofrequency field (B1) distribution, as measurements are sensitive to the subject volume size and the region-of-interest location [31]. Given this relationship, we evaluated the effect of distance on the 20 cm loop coil element of the carbon Helmholtz pair. Using a bicarbonate point phantom, we found that the TG is well described by a linear relationship with distance from the centre, and this can be taken into consideration when evaluating the relationship between the proton and carbon loading.

Given our results, we believe that a reasonable next step is the evaluation and automated optimisation of other parameters to the signal receive chain. In this study, we reduced the system analogue (R1) and digital (R2) receive gain on the proton and hyperpolarised xenon gas to avoid analogue-to-digital converter (ADC) signal overflow and possible signal clipping. The X-nuclei commonly have a low SNR compared to the proton and typically would require, if anything, an increase in the receiver gains [32]. However, hyperpolarisation greatly increases sensitivity [33,34,35], including beyond the level of proton MR, increasing the risk of ADC signal overflow and clipping. Receive gain optimisation is, therefore, more complex as it very much depends on the MR system, receive coils, sample size, and nuclear polarisation. In hyperpolarised carbon, the delivered sample dose is fixed, and we would expect R1/R2 to depend primarily on anatomy. However, in xenon gas, the subject height (as a proxy for lung volume) is often used to determine the amount of administrated xenon gas, which may therefore be used to automatically determine R1 and R2.

There are several limitations in this study. For example, this study only evaluates the relationship in sodium, xenon, and carbon and with a low number of coils. Including more nuclei and different coil designs would improve understanding of the relationship between the proton and X-nuclei TG. Nevertheless, from simulations (Appendix A.3, X-nuclei linear relationship), we find the loading of the coil (and therefore the required transmit gain) to scale with frequency, supporting the assumption as to the method’s application to other X-nuclei. Loading phantoms used in the carbon study are all part of the X-nuclei phantom package provided by the vendor. However, to adjust for change in the TG settings based on the distance between the loop coil elements, we created a bicarbonate phantom. This increases the complexity for sites in reproducing the experiments even with specifications as described in the paper. The study only evaluated the data acquired from the systems of one vendor; including more vendors and sites would increase the method’s applicability.

## 5. Conclusions

The study demonstrates a simple method to calibrate transmitted radio frequency power gain for X-nuclei using that of a proton and equations for deriving values for various coils.

## Figures and Tables

**Table 1:** Overview of pros and cons of X-nuclei transmitter setting methods.

Nuclei	Method	Pros and Cons
Hyperpolarised Helium (^3^He)	A one-time procedure using a pickup coil and a ^3^He phantom [24,25].	Pro: Easy to set up for each coil providing transmitter settings without the use of hyperpolarised helium gas. Con: Use subject weight as loading and may introduce a bias given the difference in body coil and X-nuclei loading.
Sodium (^23^Na)	Natural abundance X-nuclei prescan of or default values [8,26,27].	Pro: Sodium signal has a high natural abundance in vivo making the signal renewable. Given the low sensitivity, the addition in time is limited compared to imaging acquisition time. Con: A dedicated X-nuclei scan needs to be performed per subject introducing workflow complexity.
Hyperpolarised Carbon (^13^C)	Using phantoms (e.g., urea or bicarbonate) or historical default values [16,18,28].	Pro: Ability to set transmitter settings without the use of hyperpolarised carbon. Con: Phantoms are placed away from the region of interest, introducing a bias. Default values may vary significantly in the abdominal and thoracic regions.
Hyperpolarised Xenon (^129^Xe)	X-nuclei prescan using low-concentration hyperpolarised xenon [3,29].	Pro: Provides in vivo pulmonary calibration of the xenon gas calibration. Con: Requires administration of an additional hyperpolarised xenon gas dose.

**Figure A2:**
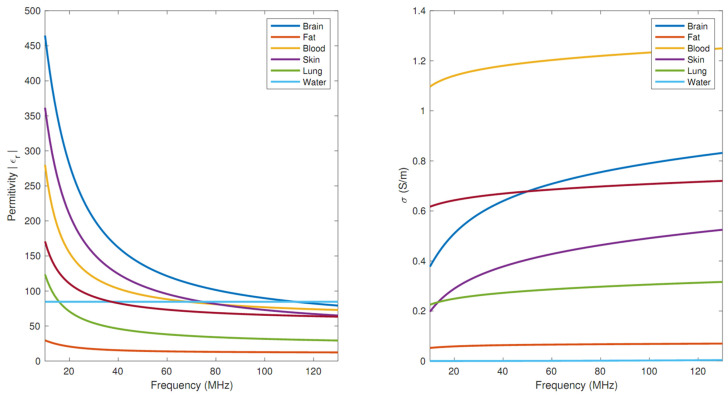
Tabulated $\left|\epsilon_{r}\right|$ and $\left|\sigma \right|$, in SI units, for a variety of human tissues.

## Data Availability

The data presented in this study are available on request from the corresponding author. The data are not publicly available due to GDPR regulations.

## Appendix A

### Appendix A.1. Study Design—Carbon Phantom Setup

The relationship between the proton and carbon TG was evaluated in phantoms. Initially, a dimethyl silicone phantom (DSP) containing natural abundance carbon [22] (GE HealthCare, Waukesha, WI, USA) was placed in the load rings, mimicking head and body loading (Figure A1). Coil distance dependency was evaluated using a 38 mm diameter spherical bicarbonate phantom containing a 1.0 M solution of ^13^C-bicarbonate sodium salt (85 mg/mL) in water. The phantom was placed 10 cm above the lower loop coil, and the distance between the coil elements was increased using cushions from 20 cm to 30 cm in intervals of 5 cm. Scanning was repeated five times by resetting shimming of B0 between the scans. The setup used is illustrated in Figure A1.

The DSP is a sphere with a diameter of 18 cm. The head-sized load ring (inner diameter = 18 cm, outer diameter = 24 cm, and length = 18 cm) and the body-sized load ring (inner diameter = 29 cm, outer diameter = 34 cm, and length = 27 cm) enclosed the DSP in the two loading experiments. The phantoms and load rings are part of the scanner system quality assurance package.

### Appendix A.2. Results—Carbon Composite Functions

Correction of distance is performed by combining the two carbon TG functions (loaded and unloaded) into a composite function. This is completed by substituting the load-varying function into the coil element distance-varying function, as seen below, where *x* equals distance.

(A1) $$g\left(x\right)=ax+b$$

(A2) $$f\left(x\right)=cx+d$$

(A3) $$f\left(g\left(x\right)\right)=c\left(ax+b\right)+d=cax+cb+d$$

(A4) $$f\left(g\left(x\right)\right)=0.266\cdot \left(0.515x+5.340\right)+10.65$$

(A5) $$f\left(g\left(x\right)\right)=0.137x+12.07$$

The composite transmit gain equation combines both loading and distance in a linear relationship.

### Appendix A.3. Discussion—X-Nuclei Linear Relationship

Although there are clearly great differences in coil design for proton and X-nuclear coils, it is worth considering how optimally tuned coils scale with frequency. It can be shown from Maxwell’s equations that, assuming biological materials have complex conductivity and permittivity and have no free charges or currents, a modified Helmholtz equation of the following form holds (and similarly for B):

(A6) $$\nabla^{2}E\left(r\right)+\omega^{2}\mu \epsilon \left(r\right)\left(1+\frac{\sigma \left(r\right)}{i\omega \epsilon \left(r\right)}\right)E\left(r\right)=0$$

And where the complex permittivity and conductivity can be related to the electric and magnetic fields within the scanner, the following holds:

(A7) $$\begin{array}{rr} \epsilon_{r}\left(r\right) & =\frac{-1}{\omega^{2}\mu \epsilon_{0}}R\left(\frac{\nabla^{2}B_{1}^{+}\left(r\right)}{B_{1}^{+}\left(r\right)}\right)=\frac{-1}{\omega^{2}\mu \epsilon_{0}}R\left(\frac{\nabla^{2}E\left(r\right)}{E\left(r\right)}\right) \end{array}$$

(A8) $$\begin{array}{rr} \sigma_{r}\left(r\right) & =\frac{1}{\omega \mu }I\left(\frac{\nabla^{2}B_{1}^{+}\left(r\right)}{B_{1}^{+}\left(r\right)}\right)=\frac{1}{\omega \mu }I\left(\frac{\nabla^{2}E\left(r\right)}{E\left(r\right)}\right) \end{array}$$

Empirically, it is found that the frequency dependence of these parameters is well parameterised in biological tissues across a frequency range spanning from 10 Hz to 20 GHz by a parametric model of the form of a modified Lorentzian with empirically determined coefficients:

(A9) $$\bar{\epsilon }=\epsilon_{\infty }+\frac{\epsilon_{s}-\epsilon_{\infty }}{1+{\left(i\omega \tau \right)}^{\left(1-\alpha \right)}}\equiv \epsilon_{\infty }+\frac{\Delta \epsilon }{1+{\left(i\omega \tau \right)}^{\left(1-\alpha \right)}}$$

which is a function of four parameters beyond the frequency of interest, ω: an individual relaxation time-constant, τ, that accounts for dipole relaxation effects analogous to magnetic hysteresis; a dimensionless ‘stretching’ parameter α and the limiting values of the real part of the polarisation, $\epsilon_{s}$ at the static-field case of ω=0 and $\epsilon_{\infty }$ as limω→∞.

Similarly, real conductivities are complex and obey a similar semi-empirical model, often described in terms of ρ rather than σ directly, where J=σE⇔E=ρJ:

(A10) $$\rho *=\rho_{\infty }+\frac{\rho_{0}-\rho_{\infty }}{1+{\left(i\frac{\omega }{2\pi F_{T}}\right)}^{\alpha }}$$

where $\rho_{0}$ and $\rho_{\infty }$ are limiting values at DC and very high frequency, respectively, and $F_{T}$ is known as the “top frequency”, the frequency at which the magnitude of the imaginary component of ρ passes its maximum. The exponent α is a parameter that depends upon the intrinsic response of cell membranes and the dispersion of the cell within a given tissue; microscopically, it models the orientation and interactions of the dipoles and capacitative responses of cell membranes.

Taken together, therefore, the dielectric response determines the coil’s sample noise as a function of frequency and effective loading, which itself is electrodynamically responsible for determining transmit gain. A tuned and loaded coil has the following:

(A11) $$Q\approx \frac{\omega L}{R}\approx \frac{\omega L}{R_{\mathrm{Coil}}+R_{\mathrm{Sample}}}$$

where $R_{\mathrm{Coil}}$ is the coil resistance, depending on its electrical construction and characteristics, and where the sample resistance depends on the patient as follows:

(A12) $$R_{\mathrm{sample}}\propto {∭}_{\mathrm{Patient}}\sigma \sigma \left(r\right){\left|E\left(r\right)\right|}^{2}\;d^{3}r$$

The ϵ and σ (or ρ) of the samples are effectively fixed by their intrinsic composition but vary as a function of both ω and r. This gives rise to approximate total scaling relationships, where we expect the loaded Q of the coil to vary with frequency as follows:

(A13) $$\begin{array}{rr} R & \propto \alpha \omega^{\frac{1}{2}}+\beta \sigma \omega^{2} \end{array}$$

(A14) $$\begin{array}{rr} \Rightarrow Q & \propto \frac{1}{\alpha \omega^{1/2}\;+\;\beta \sigma \omega^{2}}, \end{array}$$

with βσ>α for most human coils at their designed frequency.

An illustration of the typical empirical behaviour of ϵ and σ as a function of frequency is shown in Figure A2. Both are well approximated by power law behaviour with as $\omega^{\gamma }$, with γ as a parameter that varies per tissue. For that reason, we expect the loading of the coil (and therefore the required transmit gain) to scale with frequency as given above, which can be approximated linearly about a point $\omega_{0}$ as follows:

(A15) $$Q\left(\omega \right)\propto \frac{1}{\alpha \sqrt{\omega_{0}}+\beta \omega_{0}^{2+\gamma }}+\left(\omega -\omega_{0}\right)\frac{\left(-\alpha -2\beta \left(\gamma +2\right){\omega_{0}}^{\gamma +\frac{3}{2}}\right)}{2\omega_{0}^{\frac{3}{2}}{\left(\alpha +\beta \omega_{0}^{\frac{3}{2}+\gamma }\right)}^{2}}+O{\left(\omega -\omega_{0}\right)}^{2}+\ldots \;$$

This therefore justifies the approximation made in expecting a linear relationship between the proton and X-nuclear transmit gain, if the coils are sample-noise dominated; differences in body composition appear linearly in the above.
